# Examining Challenges to the Incorporation of End Users in the Design of Digital Health Interventions: Protocol for a Systematic Review

**DOI:** 10.2196/28083

**Published:** 2021-07-26

**Authors:** Anthony Duffy, Greg Christie, Sylvain Moreno

**Affiliations:** 1 School of Interactive Arts & Technology Simon Fraser University Surrey, BC Canada; 2 Digital Health Circle Surrey, BC Canada

**Keywords:** digital health, end user(s), user experience, UX, health behavior, intervention, co-design, mobile health, mHealth

## Abstract

**Background:**

The process of designing a digital health intervention (DHI)—also referred to as mobile health or eHealth—spans needs assessments, technical functionality and feasibility, user satisfaction, effectiveness, impact, and value. These interventions are causing a rapid evolution in the landscape of health care. Multiple studies have shown their propensity to extend both the quality and reach of interventions. However, failure to improve DHI design is linked to failed uptake and health outcomes. This dilemma is further conflicted by the colliding backdrops of the digital and health industries, both of which approach, understand, and involve end users differently in the framing of a DHI.

**Objective:**

The objective of this systematic review is to assess the challenges to incorporating end users in the design stage of digital health interventions, to identify key pain points, and to identify limitations and gaps for areas of future investigation.

**Methods:**

The PRISMA-P (Preferred Reporting Items for Systematic Reviews and Meta-Analysis Protocols) checklist will be used to structure this protocol. A systematic search of the PsycINFO, PubMed (MEDLINE), Web of Science, CINAHL, Scopus, and IEEE Xplore databases will be conducted. Additionally, the PerSPEcTiF guidelines for complex interventions will be consulted. Two reviewers will independently screen the titles and abstracts of the identified references and select studies according to the eligibility criteria. Any discrepancies will then be discussed and resolved. Two reviewers will independently extract and validate data from the included studies into a standardized form and conduct quality appraisal.

**Results:**

As of February 2021, we have completed a preliminary literature search examining challenges to the incorporation of end users in the design stage of DHIs. Systematic searches, data extraction and analysis, and writing of the systematic review are expected to be completed by December 2021.

**Conclusions:**

This systematic review aims to provide an effective summary of key pain points toward incorporating end users in DHIs. Results from this review will provide an evidence base for a better approach to end user involvement in the interest of improving efficacy and uptake of DHIs.

**Trial Registration:**

PROSPERO International Prospective Register of Systematic Reviews CRD42021238164; https://www.crd.york.ac.uk/prospero/display_record.php?RecordID=238164

**International Registered Report Identifier (IRRID):**

PRR1-10.2196/28083

## Introduction

### Background

Digital health interventions (DHIs), often referred to as mobile health (mHealth) or eHealth, are dynamic solutions that span needs assessments, technical functionality and feasibility, user satisfaction, effectiveness, impact, and value [1]. The domain of DHIs combines the expertise of digital and health professionals; these interventions are rapidly transforming health care, providing solutions that are emotional, decisional, or behavioral, and they are delivered with or without expert facilitation [2], bringing us closer to the dream of personalized medicine.

With one-third of individuals in the United States now using technology to manage their health, digital health revenues are expected to exceed US $500 billion by 2025 [3]. The World Health Organization declared that “people have the right and duty to participate individually and collectively in the planning and implementation of their health care” [4]. Digital health technologies (DHTs) are accelerating this vision. By enabling users to be better informed about their health, share experiences, and change perceptions (and stigmas), as well as enabling them to assess, monitor, and prioritize their health, DHIs are blending the solution space of patients and health care professionals [5].

Despite their promise, successful DHIs are challenged by a disparity in understanding and addressing users in the intervention space. A 2018 systematic review on mood disorders determined that acceptance, appropriateness, and availability framed the successful outcome of a digital solution [6]. Additionally, recent systematic reviews by Moore et al (2019) [7] and Vandekerckhove et al (2020) [8] explored forms of participatory design at the methodological level, seeking to understand current frameworks and their suitability. However, there is a need to “scratch beneath the surface” to understand and triangulate pain points toward successfully incorporating end users in DHIs at the design level.

More broadly, this approach can yield a better understanding of who the user is and how acceptance is obtained and defined. Because digital health is an inter- and transdisciplinary domain, the very definition of an end user (user/patient/human/subject) is approached differently from the perspectives of clinical, technical, and user-centered design, respectively. In an initial exploratory review of the problem space, we reviewed 54 papers. We identified a plethora of design frameworks, often with overlapping or nuancing approaches (human-centered, person-based, patient-centered, patient-led, etc), each framing the perspective of the end user differently. Nonetheless, concerns related to the uptake [9] of DHIs cut across the digital health domain regardless of the design approach. The role of incorporating the end user (the primary user of the intervention: patient, person, or sometimes practitioner) is key. This underscores the need for a systematic review to study and improve end user research in DHIs at the root level—from the perspective of the end user—exploring pain points unexclusive of a particular vantage point (health or digital) or framework.

Traditionally, within health care, randomized controlled trials (RCTs) are the gold standard approach to determining the clinical effectiveness of an intervention. Although this approach is quantitatively rigorous and statistical, it lends little to working with users (or patients) during the software development stage [10], where ideation and user feedback can be returned rapidly to ideate pivots in the intervention design. A health intervention that is proven to have clinical impact (through RCT results) will not result in uptake if users do not validate its usefulness. Contrastingly, within the digital industry, an interdisciplinary nondeterministic [11] agile approach weighs the qualitative feedback of user opinion, testing, interviews, and interaction to predict uptake [12]. Health validation also involves clinical and governmental approval [13], which challenges the laissez-faire approach to end user validation originating from mainstream app development and gaming. The health industry is rooted in rigor and research [14], validating health outcomes rather than user experiences. The thoroughness of health care juxtaposes the rapidity of agile development, creating a very different sense of how an end user should be incorporated.

The contrasting lens of what end users are and how to incorporate them is a byproduct of two different industries with two different definitions of a successful outcome. This is further complicated by the vast diversity of health stakeholders (clinicians, health experts, academics) and digital stakeholders (developers, designers, marketers, managers), which confounds the resolution space. From the health side of the room, the outcome is improved health or reduced costs, analyzed over many years. From the digital side of the room, the outcome is a digital product designed with user satisfaction in mind.

Thus, there is a clear need to understand the challenges the digital health industry faces in working with end users. Research has shown that limiting understanding of users, their needs, and the context of their solutions has induced failure in both uptake and effectiveness [15]. To this end, over the past 5 years, there have been several attempts to create agile processes tailored to the unique challenges of digital health development [13,14,16-20]. In each attempt, there is an understanding that uptake is directly connected to a successful co-design but also that typical agile industry approaches cannot simply be ported into the digital health domain unmodified, owing to the unique constraints of the health industry. The recent trend of shifting focus from user-centered to human-, person-, patient-centered, etc, to better accommodate the needs of digital health users has not eliminated the challenge to successful incorporation of end users. It is within this lens that we wish to triangulate the recurring pain points that challenge the integration of users in DHI design.

### Research Questions

The aim of the to-be-performed systematic review is to examine challenges to the incorporation of end users in the development of DHIs. The review will focus on three main questions. Firstly, how are end users currently incorporated into iterative DHI design, and what is the effectiveness of current methods? Secondly, what are the most common pain points encountered while incorporating end users in DHI design? Finally, to inform future research, what are the current limitations and gaps in end user research, such that better fluidity can be achieved in blending end users into the iterative design process in digital health, in the interest of greater efficacy and uptake?

## Methods

### Study Design

A preliminary search for existing systematic reviews on the topic of end user challenges in DHIs has been conducted in the following major databases: CINAHL, PubMed (MEDLINE), Web of Science, PsycINFO, Scopus, IEEE Xplore, PROSPERO, and the Cochrane Database of Systematic Reviews. We found no specific qualitative synthesis systematic reviews that collected the challenges in working with end users in DHIs. This systematic review protocol will follow the Cochrane Handbook for Systematic Reviews [21] and will use the PRISMA-P (Preferred Reporting Items for Systematic Reviews and Meta-Analysis Protocols) checklist for reporting the protocol [22] (the PRISMA-P flow diagram is included in Multimedia Appendix 1) [22]. This protocol is registered on PROSPERO (registration number CRD42021238164). We will follow 6 stages in this systematic review: (1) literature search, (2) article selection, (3) data extraction, (4) quality appraisal, (5) data analysis, and (6) data synthesis. This review will gather evidence on the effectiveness, challenges, and gaps in designing DHIs with end users and the opportunities for further research and development.

### Eligibility Criteria

Considering the complexity of DHIs, we have employed the PerSPEcTiF [23] guidelines for intervention (Table 1). We have selected this framework due to its suitability for qualitative synthesis in the health care domain.

**Table 1:** Details of the PerSPEcTiF framework as applied to this review.

Initial	Definition	Details
Per	Perspective	From the perspective of end users
S	Setting	In the setting of digital health
P	Phenomenon of interest/problem	What are the most prominent pain points?
E	Environment	Within an environment of designing DHIs^a^
(C)	Comparison (optional)	N/A^b^
Ti	Time/timing	During ideation and co- designing
F	Findings	In relation to understanding the challenges to successful incorporation of end users in the successful design of DHIs

^a^DHIs: digital health interventions.

^b^N/A: not applicable.

Studies will be included in this review that (1) address research on DHIs; (2) focus on interaction and co-design with end users; (3) explain results such that uptake, effectiveness, satisfaction, and health outcomes are discernable, positively or negatively; and (4) describe actionable procedures for better DHI design.

Because digital health is a rapidly evolving environment, we limited the search to studies conducted from 2015 until the date of the search commencement.

### Search Strategy

We will systematically search the following electronic databases: PsycINFO, PubMed (Medline), Web of Science, CINAHL, IEEE Xplore, and Scopus. We have selected these databases according to preliminary searches and consultation with experts and librarians in this field. Keywords related to digital health interventions will be used. We will adapt the search strategy as needed to return a breadth of papers without retrieving an unmanageably large number of irrelevant articles.

A draft of the search terms that will be used in this review are grouped into three categories in Table 2.

The three clusters will be searched individually to compare and compile results from three different vantage points in digital health:

- Cluster 1: Health behavior (owing to behavior changes in health care resulting from digital health interventions)
- Cluster 2: User experience (owing to engineering, development, and design in digital health)
- Cluster 3: Methodologies and frameworks (owing to digital project facilitation and to health care intervention design and policy)

**Table 2:** Search terms and strings to be used in the systematic review.

Category	Keyword
Health behavior	“digital health” or “mHealth^a^” or “eHealth” and “end user*” or “end-user*”
User experience	“user experience” or “UX^b^” or “user centred” or “user centered” or “human centred” or “human centered” or “patient centred” or “patient centered” or “person based” or “person centered” or “person centred” or “participatory design” or “involvement” and “digital health” or “mHealth” or “eHealth”
Methodologies and frameworks	“agile” or “scrum” or “kanban” and “digital health” or “mHealth” or “eHealth”

^a^mHealth: mobile health.

^b^UX: user experience.

### Inclusion Criteria

The primary criteria for inclusion will be (nonsingle) case studies; observational studies, including cross-sectional surveys; cohort studies; qualitative studies; and nonrandomized studies (before-and-after studies, interrupted time series studies). Only English language studies will be included. Due to the rapid innovation of digital health, only studies from January 1, 2015, to the date of the search commencement will be included. Any population group, geographical location, or topic that influences end user interaction in DHIs will be considered. There must, however, be an output that critically analyses the involvement of a user (inclusive of like terms: patient, person, human) in a DHI.

### Exclusion Criteria

We will exclude studies that are not published in English. We will exclude single user/patient studies.

### Screening and Article Selection

All articles identified and selected from the database searches will be stored in the reference management software EndNote (Clarivate Analytics), which will be used to eliminate duplicates, and tag and organize the research structure. Two independent reviewers will screen the titles and abstracts of all the studies. The full text of the remaining articles will then be examined to determine final eligibility. A PRISMA flow diagram will be used to record the details of the screening and selection process so that the study can be reproduced.

### Data Extraction

To collate the results from qualitative studies on DHI end user challenges, we will extract data from studies that meet our inclusion criteria. Using a spreadsheet, the following data will be extracted: (1) title; (2); authors; (3) year of publication; (4) source of data; (5) country of study; (6) study characteristics (design, aim, population, primary user type); (7) outcomes (health outcomes, usability, user experience, feasibility, resource implication [including cost]); (8) limitations (functional, user-reported, health outcomes, potential improvements). As a pilot, we will extract data from a small number of studies before refining the final data extraction form. We will contact the authors and collaborators of publications to clarify data and feedback where necessary. Two reviewers will review the full text of all the papers included in the final section. Extracted data will be reviewed by one additional reviewer. Disagreements will be resolved by consensus discussion.

### Quality Appraisal and Risk of Bias Assessment

Once the final selection of studies has been made, two independent reviewers will assess the risk of bias for included studies. If disagreement in judgment occurs, the reviewers will discuss disagreements before consulting a third reviewer. Because the majority of included papers are expected to be qualitative rather than quantitative (nonrandomized intervention assessment), we will use the Risk Of Bias in Non-randomized Studies of Interventions (ROBINS-I) tool [24]. All papers will be assessed independently by two reviewers relative to the PerSPEcTiF guidelines in the eligibility criteria. A table will be created to summarize risk of bias graded as high, moderate or low. 

### Data Analysis and Synthesis

It is unlikely that a meta-analysis will be feasible due to the anticipated variety of study aims, methods, and reported outcomes. Therefore, a narrative synthesis will be performed to describe and summarize the identified studies. Rather than a statistical analysis, we will provide a structured narrative and/or summarized tables. The data synthesis will help assess areas of strength and weakness in both inter- and transdisciplinary approaches to working with end users in the design of DHIs, specifically identifying key pain points and suggesting approaches to resolve them. Finally, the identification of limitations and gaps in research will help establish a direction for the improvement of future DHIs in both efficacy and uptake.

## Results

As of February 2021, we have completed a preliminary literature search examining challenges to the incorporation of end users in the development of DHIs. Systematic searches, data extraction and analysis, and writing of the systematic review are expected to be completed by December 2021.

## Discussion

This systematic review will provide a systematic, transparent review of the literature to better understand the most prominent pain points encountered when integrating end users into the iterative design of DHIs. In this section, any researcher assumptions will also be discussed, as well as the conclusiveness of the data; limitations of the systematic review; gaps in the current literature; and possibilities for future research.

The three main contributions of our review will be the following: (1) investigation into current DHI end user incorporation and its strengths and weaknesses; (2) detailing and triangulating the most common pain points encountered in seeking to incorporate end users in iterative design; (3) a discussion on the current limitations, gaps, and methods to inform better research toward a more fluid approach to incorporating end users in DHIs. Any amendments or modifications made in the protocol will be outlined and reported in the final papers. We anticipate that a limitation of this study will be the lack of quantitative studies available to cross-validate determinations made from qualitative studies. This is, fundamentally, a byproduct of the digital health arena, in which technological innovation outpaces long-term study timelines. Additionally, the restriction of studies published from January 1, 2015, forward, albeit by design (to focus on cutting-edge approaches), limits historical context to emerging DHI end user approaches. Based on the data synthesized, the key implications drawn will be discussed toward better design, incorporation, and evaluation of end user involvement in DHIs moving forward.

## Appendix

Multimedia Appendix 1 PRISMA-P (Preferred Reporting Items for Systematic Reviews and Meta-Analysis Protocols) flow diagram.

## Abbreviations

DHI: digital health intervention
DHT: digital health technology
mHealth: mobile health
PD: participatory design
PRISMA-P: Preferred Reporting Items for Systematic Review and Meta-Analysis Protocols
RCT: randomized controlled trial
ROBINS-I: Risk Of Bias in Non-randomized Studies of Interventions
